# An Efficient and Robust Dual-Channel Signal Gluing Method for Atmospheric Lidar

**DOI:** 10.3390/s25185807

**Published:** 2025-09-17

**Authors:** Tong Wu, Kai Zhong, Xianzhong Zhang, Fangjie Li, Xinqi Li, Guxi Chen, Degang Xu, Jianquan Yao

**Affiliations:** 1 School of Marine Science and Technology, Tianjin University, Tianjin 300072, China; wutong_101@tju.edu.cn; 2 Key Laboratory of Optoelectronic Information Science and Technology (Ministry of Education), School of Precision Instruments and Opto-Electronics Engineering, Tianjin University, Tianjin 300072, China; zxzhitr@tju.edu.cn (X.Z.); lifangjie@tju.edu.cn (F.L.); qixinli19990605@tju.edu.cn (X.L.); cgx_2023202233@tju.edu.cn (G.C.); xudegang@tju.edu.cn (D.X.); jqyao@tju.edu.cn (J.Y.)

**Keywords:** lidar, dual-channel signal gluing, improved whale optimization algorithm (IMWOA), entropy weight method (EWM)

## Abstract

Lidar serves as a vital active remote sensing instrument for exploring the atmosphere. However, the detection range of lidar is significantly constrained by the dynamic range of photo-detectors. To mitigate this limitation, atmospheric lidars are commonly equipped with multiple channels to capture signals from different altitude ranges, making the high-quality gluing of multi-channel echo signals crucial for accurate data retrieval. In this paper, an efficient dual-channel signal gluing method based on the improved whale optimization algorithm (IMWOA) and the entropy weight method (EWM), named IMWOA-EWM, was proposed. Here, the IMWOA method was used to optimize the fitness function, achieving higher computational efficiency. The weights of the correlation coefficient *R*, regression stability coefficient *S* and mean fit deviation *D* were determined using EWM, which together constitute the fitness function. Through signal gluing experiments conducted with ground-based aerosol lidar data, IMWOA-EWM can accurately identify the optimal gluing region, due to IMWOA’s excellent global search capability and the higher weight assigned to the objective function *S* by EWM. Meanwhile, regarding computational efficiency, its runtime is only half that of IGWO-RSD. Additionally, the applicable conditions of the weights in IMWOA-EWM were explored, which indicate that IMWOA-EWM has good robustness for atmospheric lidar signal gluing.

## 1. Introduction

Lidar has become an indispensable instrument for accurately measuring various atmospheric properties, e.g., aerosols, wind fields, density, and temperature, due to its high spatial and temporal resolution [1]. However, the intensity of the lidar echo signal decreases inversely with the square of the distance. In medium- and long-range detection, this attenuation can result in a dynamic range exceeding six orders of magnitude, surpassing the linear response capability of state-of-the-art single-photon detectors [2]. To resolve this issue, atmospheric lidar systems typically employ multi-channel techniques to capture signals from different altitude ranges.

There are two main approaches to achieve multi-channel detection. The first approach utilizes the circuit separation technique to split the signal from a single detector. Different detectors working in photon counting mode (PC) and analog-to-digital mode (AD) are then used to collect data from far- and near-field channels, respectively [3]. This approach enhances the detection of weak signals at high altitudes. The second approach involves separating signals of different intensities in the receiving system, which can be accomplished by using multiple receiving telescopes with different apertures [4,5] or by employing a beam splitter [6,7,8]. Among these, the optical architecture based on a beam splitter offers high stability, low cost, and insensitivity to environmental variations, gradually evolving into one of the mainstream structures for multi-channel detection.

The high-quality gluing of multi-channel echo signals prior to inversion can effectively extend the usable detection range, thereby supporting accurate parameter retrieval. For this purpose, linear regression should be carried out over a specific height range of the multi-channel signals to derive the fitting coefficients. Subsequently, the glued signal is acquired through either point-by-point or piecewise gluing mode. Previous studies are dedicated to identifying the optimal gluing region. These efforts primarily involve three methods: strength constraint, altitude constraint, and constraint based on specific evaluation indicators. The strength constraint refers to the upper limit of the fitting signal strength determined by the nonlinearity of the detectors and the lower limit determined by the SNR of the near-field channels [9,10]. However, Zhang et al. found that as the correlation of the dual-channel signal decreases, the fitting results become unstable [11]. Therefore, D’Amico et al. achieved altitude constraints by gradually reducing the height range with a fixed step size, using the correlation coefficient and the regression stability coefficient as evaluation indicators [12]. Huang et al. adopted the correlation coefficient and gluing deviation per kilometer as evaluation indicators and conducted fixed-step traversal within the given height range [5,13]. Both works offered relatively credible gluing regions, but it is worth noting that iteration with a fixed step size is comparatively mechanical and limits the ability to search for the optimal gluing region. Additionally, gluing methods by means of the statistical principles [14], lamp mapping technique [15], and spatiotemporal variance [16] have been proposed. However, these methods are very complex to operate or have limited applicability.

Inspired by advanced optimization techniques, Duan et al. proposed a hybrid algorithm that combines the non-dominated sorting genetic algorithm II (NSGA-II) with the neighborhood rough set (NRS) to effectively identify the optimal solution in randomly generated gluing regions, named NRSWNSGA-II [17]. In NRSWNSGA-II, the correlation coefficient *R*, regression stability coefficient *S*, and standard gain ratio deviation *T* were designed to form the fitness function, which showed better gluing accuracy and stability than the other methods. However, its fitness function and convergence ability could still be improved. In 2023, Li et al. suggested substituting *T* with the mean fit deviation *D* to construct the fitness function together with *R* and *S* and replacing NSGA-II with the improved gray wolf optimizer (IGWO), which formed the IGWO-RSD method. This improvement not only reduces the dependence on prior information but also significantly enhances the algorithm’s convergence speed and solution accuracy [18].

The IGWO-RSD performs remarkably well but has certain limitations. For example, the high computational complexity of IGWO leads to a longer runtime. Additionally, the weights of *R*, *S*, and *D* are obtained by training with NRS. Thousands of training samples are required to achieve stable weights, which further increases the time overhead.

To address these issues, this paper focuses on developing an efficient signal gluing method for dual-channel atmospheric lidar. A hybrid gluing method based on the improved whale optimization algorithm (IMWOA) and the entropy weight method (EWM), named IMWOA-EWM, was proposed. Here, IMWOA can effectively improve computational efficiency compared with IGWO. The fitness function of IMWOA was composed of *R*, *S*, and *D*, with weights allocated using EWM. Compared to NRS, EWM does not require excessive training samples, thereby significantly reducing computational load. The runtime, solving capability, gluing accuracy, and stability of IMWOA-EWM were verified by gluing the ground-based aerosol lidar data measured by the Arctic high spectral resolution lidar (AHSRL), which was developed by the University of Wisconsin in Madison, USA [19,20]. Moreover, by conducting signal gluing experiments with full-day signals collected by the AHSRL system in Korea and Singapore, the transferability and applicable conditions of the weights in IMWOA-EWM were explored.

The main contributions of this paper are summarized as follows:

1. To address the issue that the complicated design of the advanced IGWO-RSD method leads to long computation and training times, a hybrid and efficient gluing method based on IMWOA and EWM, named IMWOA-EWM, was proposed.
2. IMWOA-EWM innovatively adopts the IMWOA optimizer, which integrates advanced techniques such as the nonlinear convergence factor, flight strategy, and optimal neighborhood perturbation. Compared with IGWO utilized in IGWO-RSD, it not only achieves superior global search capabilities but also simplifies predatory behavior and reduces computational complexity.
3. Another key innovation of IMWOA-EWM is the use of the EWM method for weight allocation, which notably requires fewer training samples. This significantly reduces the computational load and time overhead compared to the NRS method employed in IGWO-RSD.
4. By conducting signal gluing experiments with full-day signals, the robustness and applicable conditions of IMWOA-EWM were discussed, providing guidelines for practical applications.

This paper is organized as follows. Section 2 presents the theoretical background, including the basic idea of signal gluing based on IMWOA-EWM, a brief review of principles of IMWOA and EWM, and the main construction of the fitness function. Section 3 describes the experimental validation of the runtime, solving capability, gluing accuracy, and stability of IMWOA-EWM. Section 4 analyzes the transferability and applicable conditions of the weights in IMWOA-EWM based on full-day gluing experiments. Finally, the conclusions are summarized in Section 5.

## 2. Materials and Methods

### 2.1. Basic Idea

**Figure 1:**
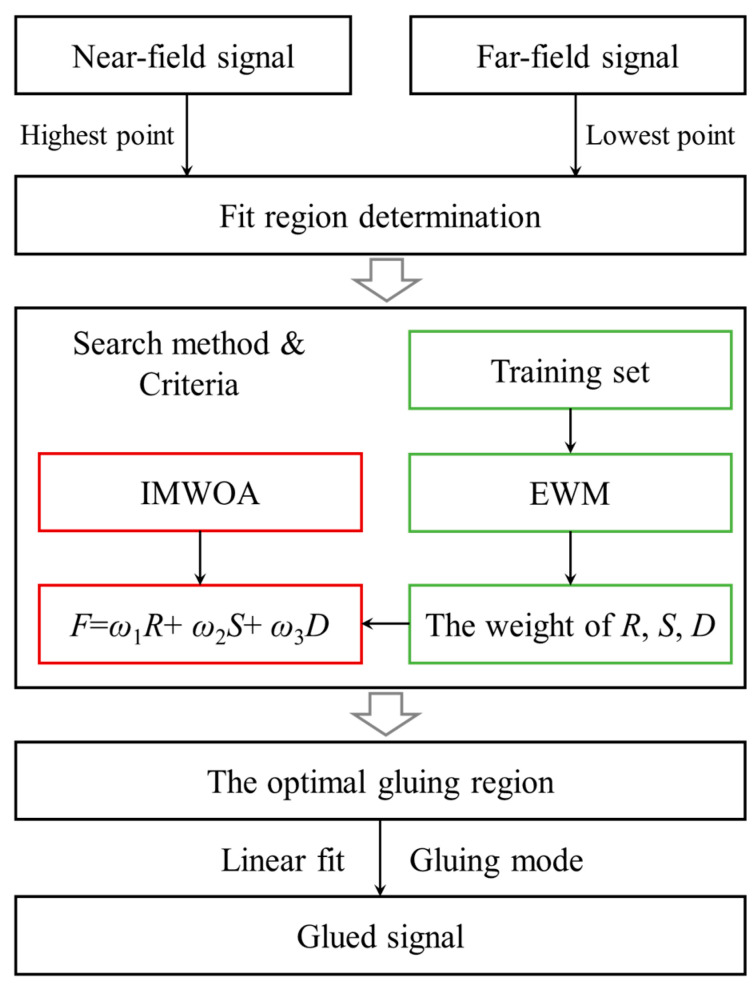
Flowchart of signal gluing based on IMWOA-EWM.

The basic idea of signal gluing is to determine a fit region in vertical altitude, where both the near-field channel signal and far-field channel signal have good signal-to-noise ratios (SNR). Then, an optimization algorithm named IMWOA-EWM is adopted to automatically search for a small part of the fit region as the optimal gluing region. The IMWOA-EWM algorithm consists of an optimization algorithm based on IMWOA and a weight allocation method based on EWM. The fitness function is formed by the fit correlation coefficient *R*, the regression stability coefficient *S,* and the mean fit deviation *D*. Finally, according to the optimal gluing region, the near-field channel signal and far-field channel signal are glued. The flowchart of signal gluing based on IMWOA-EWM is shown in Figure 1.

In the initial fit region, the lowest point corresponds to the saturation-correctable altitude of the far-field channel signal using the non-paralyzable model [12], while the highest point is determined by the maximum height at which the SNR of the near-field channel signal is greater than 10. The SNR is calculated as shown in Equation (1), where *N^near^* indicates the near-field channel signal, and *N_bg_* is the background noise. After determining the optimal gluing region [*z*_1_, *z*_2_], the near-field and far-field channel signals are fitted within this region. These fitting coefficients are then used to construct the glued signal. As shown in Equation (2), the glued signal *N_fin_* can be divided into three parts. From the ground to the lower boundary *z*_1_ of the optimal gluing region, the near-field channel signal is adopted to leverage the advantage of the low detection dead zone. In the optimal gluing region, the average of the near-field channel signal *N^near^* and the fitted far-field channel signal *N^far_fit^* is utilized to smooth the glued signal. Here, *N^far_fit^* = *KN^far^* + *b*, where *K* and *b* are the fitting coefficients, and *N^far^* represents the far-field channel signal. Above the upper boundary *z_2_* of the optimal gluing region, the fitted far-field channel signal is employed to take advantage of the high SNR. Therefore, the glued signal can effectively extend the detection range.

(1) $$SNR=\frac{N^{near}-N_{bg}}{\sqrt{N^{near}}},$$

(2) $$N_{fin}=\left\{\begin{array}{l} N^{near},z<z_{1}\\ \frac{1}{2}\left(N^{near}+N^{far\_fit}\right),z\in \left[z_{1},z_{2}\right]\\ N^{far\_fit},z>z_{2} \end{array}\right.,$$

### 2.2. Improved Whale Optimization Algorithm

**Figure 2:**
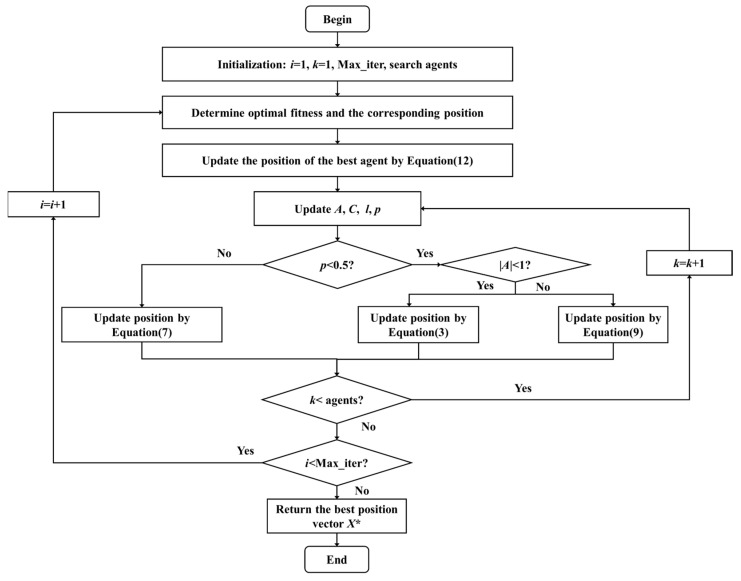
The flowchart of IMWOA.

Optimization algorithms are used to find the optimum solution under specific constraints, which have been widely applied in diverse scientific fields [21]. Inspired by the whale’s bubble network attack strategy, the whale optimization algorithm (WOA) was proposed by Mirjalini and Lewis in 2016 [22]. However, WOA is prone to becoming trapped in local optima and missing the global optimum. To address this issue, an improved whale optimization algorithm (IMWOA) has been developed by introducing a nonlinear convergence factor, optimal neighborhood perturbation, and flight strategy [23]. The flowchart of IMWOA is summarized in Figure 2, and the mathematical model of IMWOA is mainly divided into three stages: encircling prey, spiral bubble-net attack, and search for prey.

Supposing that a candidate solution is considered as the prey position, a humpback whale’s encircling of prey can be expressed as

(3) $$\vec{D}=\left|C\cdot {\vec{X}}_{i}^{*}-{\vec{X}}_{i}\right|,$$

(4) $${\vec{X}}_{i+1}={\vec{X}}_{i}^{*}-A\cdot \vec{D},$$

(5) A=2a⋅r−a,

(6) $$a=2\cdot sin\left[\frac{\pi }{2}\left(1-\frac{i}{T_{max}}\right)\right],$$

where ${\vec{X}}_{i}$ denotes the whales’ position vector, ${\vec{X}}_{i}^{*}$ is the best prey position vector, *i* indicates the current iteration, *r* is a random number in [0, 1], and *T_max_* is the maximum number of iterations. *A* and *C* are coefficients, and *A* is used to regulate the global and local search capabilities, which can be calculated by the nonlinear convergence factor *a*. As shown in Equation (6), *a* is large and changes slowly in the early stage, which can enhance the global optimization performance. Then, it decreases rapidly in the later stages of the algorithm to improve the convergence speed and accuracy.

Besides encircling prey, the mathematical model is assumed to have a 50% probability of using a spiral model to update the whale’s position during the optimization process, shown by

(7) $${\vec{X}}_{i+1}=\left\{\begin{array}{cc} {\vec{X}}_{i}^{*}-A\cdot \vec{D} & p<0.5\\ \vec{D^{\prime }}\cdot e^{bl}\cdot \cos\left(2\pi l\right)+{\vec{X}}_{i}^{*} & p\geq 0.5 \end{array}\right.,$$

(8) $${\vec{D}}^{\prime }=\left|{\vec{X}}_{i}^{*}-{\vec{X}}_{i}\right|,$$

where ${\vec{D}}^{\prime }$ represents the distance between the current whale and the best solution acquired so far, *p* is a probability number in [0, 1], the constant *b* = 1 controls the shape of the logarithmic spiral, and *l* = *n*_0_−*2i*/*T_max_* is the linear control factor that determines the step length of spiral updating, where *n*_0_ is a random number in [−1,1].

Humpback whales also randomly search for prey. If |*A*| ≥ 1, the position of a search agent will be updated according to a randomly chosen search agent ${\vec{X}}_{rand}$. With the help of lévy flights, the search agent is selected by an arbitrary step size, which is generally small but occasionally large [24]. The mathematical equation is described as

(9) $${\vec{X}}_{i+1}={\vec{X}}_{i}+\alpha_{1}⊕Levy\left(\beta \right),$$

(10) $$\alpha_{1}⊕Levy\left(\beta \right)\sim 0.01\cdot s\cdot A\cdot \left({\vec{X}}_{i}-C\cdot {\vec{X}}_{rand}\right),$$

where *α*_1_ is the step-size coefficient with a value of 0.01, and *s* represents a random step size generated by the Mantegna method, given by

(11) $$s=\frac{u}{{\left|v\right|}^{1/\beta }},$$

where $u\sim N\left(0,\sigma_{u}^{2}\right)$, $v\sim N\left(0,1\right)$, $\sigma_{u}^{2}\sim {\left(\Gamma \left(1+\beta \right)sin\left(\pi \cdot \frac{\beta }{2}\right)/\Gamma \left(\frac{1+\beta }{2}\right)\cdot \beta \cdot 2^{\frac{\beta -1}{2}}\right)}^{1/\beta }$, *β* = 1.5, and Γ denotes the standard Gamma distribution function.

To find possible optimal solutions around the current optimal agent, an external random perturbation mechanism is added in IMWOA, as shown in the following equation.

(12) $${\vec{X}}_{i}^{s}={\vec{X}}_{i}^{*}+\vec{r1},$$

(13) $${\vec{X}}_{i}^{*}=\left\{\begin{array}{c} \;{\vec{X}}_{i}^{s},\;F\left({\vec{X}}_{i}^{s}\right)<F\left({\vec{X}}_{i}^{*}\right)\\ {\vec{X}}_{i}^{*},\;F\left({\vec{X}}_{i}^{s}\right)\geq F\left({\vec{X}}_{i}^{*}\right) \end{array}\right.,$$

where $\vec{r1}$ is a random vector in [−50,50], and *F* is the fitness function. If the generated position has a smaller fitness, it is replaced with the original position. Conversely, the optimal position remains unchanged.

### 2.3. Fitness Function

Selecting an appropriate fitness function is essential for achieving the optimal solution to a specific problem. To obtain the optimal gluing region, three objective functions, *R*, *S*, and *D,* are chosen to construct the fitness function *F*, expressed as

(14) $$F=\omega_{1}R+\omega_{2}S+\omega_{3}D,$$

where *ω*_1_, *ω*_2_, and *ω*_3_ are the weights assigned to *R*, *S*, and *D*, respectively. These weights are determined by the weight allocation algorithm based on EWM, as introduced in Section 2.4.

*R* is the correlation coefficient of the linear-least-squares fit between the near- and the far-field signals. The range of *R* is [0, 1], where a higher value of *R* denotes stronger correlations between the dual-channel signals. If *R* is less than 0.9, the dual-channel signals are not linearly correlated. The expression for *R* is given by Equation (15), where *N*^near^ and *N*^far^ are the near- and far-field signals.

(15) $$R=\frac{\sum_{i}\left(N_{i}^{near}-\bar{N^{near}}\right)\left(N_{i}^{far}-\bar{N^{far}}\right)}{\sqrt{\sum_{i}{\left(N_{i}^{near}-\bar{N^{near}}\right)}^{2}\sum_{i}{\left(N_{i}^{far}-\bar{N^{far}}\right)}^{2}}},$$

If the gluing region is large, there could be a difference between the upper and lower halves of the region. The regression stability coefficient *S* was defined through regression analysis, as presented in [12]. The gluing region is divided into two equal sub-regions named *j*_1_ and *j*_2_. By solving each region using Equations (16)–(17) and performing the linear-least-squares fit on the dual-channel signals, the fitting slopes *K*_1_, *K*_2_, and the corresponding residuals *Re*_1_, *Re*_2_ are obtained. Then, the slopes *K_Re_*_1_ and *K_Re_*_2_ of the residuals over the range *z* can be calculated through Equation (18). Finally, the regression stability coefficient *S* is expressed by Equation (19), where Δ*K_Re_*_1_ and Δ*K_Re_*_1_ are the standard deviations of the slopes *K_Re_*_1_ and *K_Re_*_1_, respectively. A larger value of *S* reflects greater instability in the regression analysis results of the two regions.

(16) $$N_{j}^{near}=KN_{j}^{far},$$

(17) $$Re_{j}=K_{j}N_{j}^{far}-N_{j}^{near},$$

(18) $$Re_{j}=K_{Re_{j}}z(j=1,2),$$

(19) $$S=\frac{\left|K_{Re_{1}}-K_{Re_{2}}\right|}{\sqrt{\Delta K_{Re_{1}}^{2}+\Delta K_{Re_{2}}^{2}}},$$

The objective function *D* is defined as the average deviation between the near-field signal (*N*^near^) and the fitted far-field signal (*N*^far_fit^) for each bin within the initial fit region, as given by Equation (20). The gluing coefficients are obtained by the linear-least-squares fit between the dual-channel signals in the gluing region [*z*_1_, *z*_2_]. A larger *D* demonstrates that the deviation between dual-channel signals is greater.

(20) D=∑iNinear−Nifar_fitNinearBinnum,

where *Bin_num_* is the number of bins in the initial fit region, and *N_i_*^near^ represents the *N*^near^ in the *i*-th bin.

It should be noted that *R*, *S*, and *D* are derived from the signal itself rather than any other prior information, and therefore, no additional error is introduced.

### 2.4. Entropy Weight Method

The entropy weight method (EWM) can quantify and synthesize multiple indicators to facilitate decision-making [25], which has the characteristics of objectivity and adaptability. The specific steps for applying EWM to calculate the weights are as follows:

1. Construct a joint decision evaluation matrix **X** with *n* samples and *m* attribute indexes. Among them, *x_ij_* indicates the *j*-th attribute value of the *i*-th sample. In this paper, *m* = 3 means that there are three objective functions as attribute indexes.
  (21) $$X=\left[\begin{array}{cccc} x_{11} & x_{12} & \ldots & x_{1m}\\ x_{21} & x_{22} & \ldots & x_{2m}\\ \vdots & \vdots & \ddots & \vdots \\ x_{n1} & x_{n2} & \ldots & x_{nm} \end{array}\right],$$
2. Standardize the matrix **X** to eliminate the dimensional and unit differences among the data. The standardized matrix is denoted by **Y**, where *y_ij_* signifies the standardized value corresponding to *x_ij_*.
  (22) $$y_{ij}=\frac{x_{ij}-\min\left(x_{1j},\ldots ,x_{nj}\right)}{\max\left(x_{1j},\ldots ,x_{nj}\right)-\min\left(x_{1j},\ldots ,x_{nj}\right)},$$
  (23) $$Y=\left[\begin{array}{cccc} y_{11} & y_{12} & \ldots & y_{1m}\\ y_{21} & y_{22} & \ldots & y_{2m}\\ \vdots & \vdots & \ddots & \vdots \\ y_{n1} & y_{n2} & \ldots & y_{nm} \end{array}\right],$$
3. Calculate the specific gravity *P_ij_* of *y_ij_*, which can be defined as
  (24) $$P_{ij}=\frac{y_{ij}}{\sum_{i=1}^{n}y_{ij}},$$
4. Compute the weight coefficient *ω_j_* of the *j*-th attribute component based on its entropy value *e_j_* and difference coefficient *g_j_*, shown by
  (25) $$e_{j}=-\frac{\sum_{i=1}^{n}P_{ij}\ln\left(P_{ij}\right)}{\ln\left(n\right)},$$
  (26) $$g_{j}=1-e_{j},$$
  (27) $$\omega_{j}=\frac{g_{j}}{\sum_{j=1}^{m}g_{j}}$$

## 3. Results

### 3.1. Data Description

In this paper, gluing experiments were conducted based on a total of 8690 samples collected from measurements in Korea and Singapore. These measurements were taken by the Arctic high spectral resolution lidar (AHSRL), which was developed by the University of Wisconsin in Madison, USA. All samples were obtained with a temporal resolution of 1 h and a vertical resolution of 7.5 m, which can be downloaded from the website of the LIDAR Group at the University of Wisconsin [20]. The measured data provided by the AHSRL system has undergone preprocessing, including dead-time correction and trigger delay correction, and can be directly used for weight allocation and signal gluing.

The weights of *R*, *S*, and *D* were acquired using the EWM algorithm with 500 randomly selected samples from Korea for weight allocation. The EWM method does not rely on sample size when obtaining weights, but it is sensitive to outliers. To eliminate outliers, the median absolute deviation method [26] was applied before data standardization. Based on our team’s previous work [23], it was found that the number of search agents influences the convergence behavior. Although the results exhibit some fluctuation with varying agent counts, a size of 40 was found to already provide strong global search performance. To ensure stability, this study uses 50 search agents. Subsequently, the number of iterations was initially set to 100, but experiments showed that full convergence was consistently achieved by 50 iterations. Considering that redundant iterations are unnecessary, the number of iterations was ultimately determined to be 50. The operating parameters of IMWOA-EWM are shown in Table 1.

### 3.2. Performance Comparison of Optimizers

**Figure 3:**
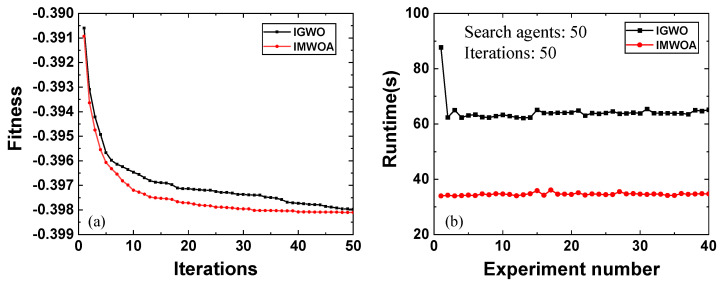
Performance comparison of optimizers, where the black curve represents the results of IGWO optimizer and the red curve indicates the results of IMWOA optimizer. (**a**) Comparison of the solving abilities of the optimizers. (**b**) Runtime comparison of the optimizers (experimental setup: Operating system: Windows, Software: MATLAB (version: R2021b), CPU: i7 processor (Intel, Santa Clara, CA, USA), GPU: RTX 4050 (NVIDIA, Santa Clara, CA, USA), RAM: 16 GB (Samsung, Suwon, Republic of Korea)).

A primary distinction between IMWOA-EWM and IGWO-RSD is the choice of the optimizer, where it is the IMWOA for IMWOA-EWM and IGWO for IGWO-RSD. Selecting an appropriate optimizer for a given problem can greatly enhance the performance of the optimization algorithm. The midday sample from Korea (12:00 to 13:00 on January 5, 2017) is characterized by strong solar background noise, which leads to significant saturation at lower altitudes. This sample is both typical and challenging. Therefore, to verify the superiority of IMWOA over IGWO in solving the optimal gluing region, the solving abilities and runtime of the two optimizers were compared based on the midday sample. The comparative experiments were conducted under the Windows operating system, with programs run using MATLAB (version: R2021b). The configuration of the computer used is an RTX 4050 GPU (NVIDIA, Santa Clara, CA, USA), an i7 processor (Intel, Santa Clara, CA, USA), 16 GB of RAM (Samsung, Suwon, Republic of Korea), and a 1 TB SSD (Western Digital, San Jose, CA, USA). The results are shown in Figure 3. In the comparison of the two optimizers, we conducted gluing experiments 40 times. During each experiment, the IGWO optimizer also performed 50 iterations with the number of search agents set to 50. The fitness function comprises *R*, *S*, and *D*, with respective weights of 0.3952, 0.2984, and 0.3064. The convergence curves of the two optimizers were generated by averaging the fitness function values across all experiments for each iteration. The horizontal axis presents the number of iterations, while the vertical axis indicates the average fitness function value. The runtime was determined by measuring the duration of each gluing experiment, with the horizontal and vertical axes representing the number of experiments and time, respectively.

As depicted in Figure 3a, IGWO tends to fall into the local optimum after 15 iterations, whereas IMWOA continues to search for the global optimal solution. This behavior can be attributed to the incorporation of a nonlinear convergence factor, flight strategy, and optimal neighborhood perturbation in IMWOA. These improvements enable the algorithm to expand the search space in the early stages, thereby enhancing global search capabilities and avoiding local optima. As shown in Figure 3b, the average runtime of IGWO is 64.33 s, while that of IMWOA is only 34.61 s, approximately half of IGWO. Moreover, the runtime of IMWOA is more stable, as evidenced by the curve. Therefore, IMWOA is more efficient than IGWO and is better suited to address the problem of identifying the optimal gluing region of multi-channel signals in atmospheric lidar.

### 3.3. Verification of Gluing Performance

**Figure 4:**
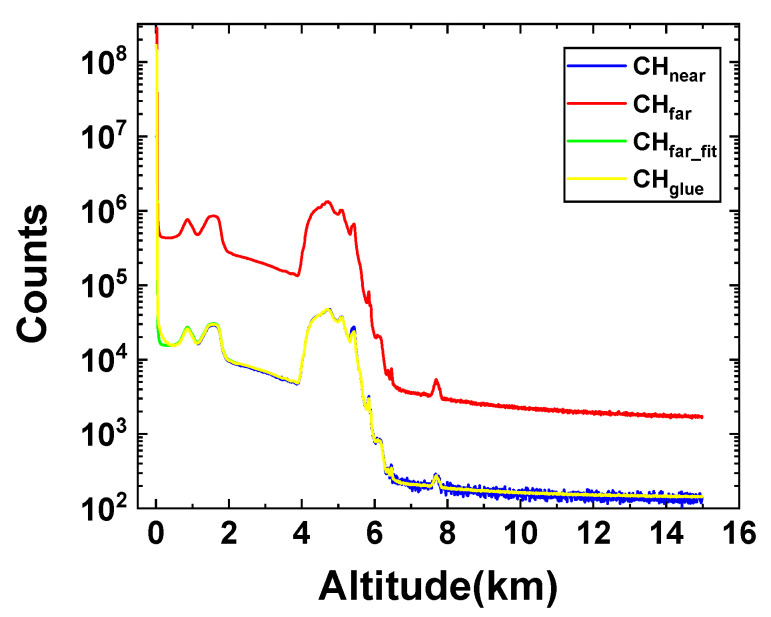
The IMWOA-EWM gluing result, where the blue curve indicates the near-field channel signal, the red curve represents the far-field channel signal, the green curve is fitted from the far-field channel signal by IMWOA-EWM, and the yellow curve represents the glued signal.

**Figure 5:**
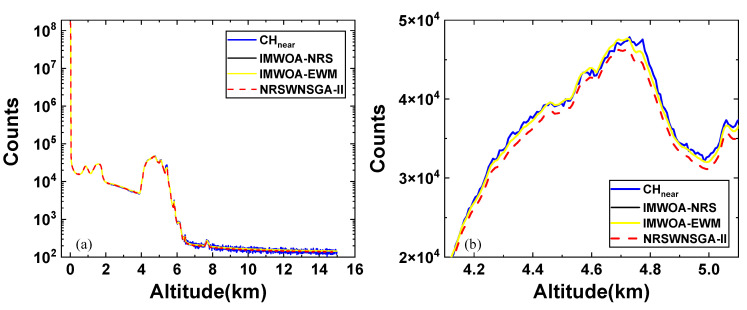
Comparison of midday signal gluing, where the blue curve represents the near-field channel signal, the black curve is glued signal obtained by IMWOA-NRS, the yellow curve shows glued signal obtained by IMWOA-EWM, and the red dashed line indicates glued signal obtained by NRSWNSGA-II. (**a**) Global plot where CH_near_ represents the near-field channel signal. (**b**) Enlarged view of the gluing results in the altitude range of 4–5 km.

To validate the gluing performance of the IMWOA-EWM method, the midday sample employed in the previous section was utilized again. As shown in Figure 4, the far-field channel signal was used to fit the near-field channel signal, and the gluing result generally matched the near-field signal well. To compare with other methods, the midday sample was glued using NRSWNSGA-II and IMWOA-EWM, respectively. Given that the IMWOA method has been proven to be more efficient and suitable than IGWO in Section 3.2, the gluing result obtained with the IMWOA optimizer and NRS weighting (referred to as IMWOA-NRS) was also compared to assess the impact of the EWM method on the gluing process. All the optimizers performed 50 iterations, and the number of search agents was set to 50. The weights of *R*, *S,* and *D* acquired by the NRS method are 0.4102, 0.2233, and 0.3665, respectively. The optimal gluing regions for the three gluing algorithms were all determined by the minimum fitness function values gained from 40 repeated experiments. The gluing results are shown in Figure 5.

As shown in Figure 5, the fitting results of IMWOA-NRS and IMWOA-EWM are consistent and match the near-field channel data well overall. This is because both methods use IMWOA as the optimizer and construct their fitness functions based on *R*, *S*, and *D*. Although the two methods employ different weight allocation strategies, the generated weights can both effectively guide IMWOA to find the optimal solution. However, EWM does not require a large number of samples for training, which considerably reduces the computational load. In the altitude range of 4–5 km, the signals at certain peaks do not match well, primarily due to the combined effects of detector saturation and cloud scattering. In fact, it is difficult to completely eliminate the errors in the near-field signals, given the limited applicability of the non-paralyzed model used for correcting saturated signals [12]. The signals fitted by NRSWNSGA-II exhibit significant deviations due to the influence of inaccurate prior information on *T* in the fitness function. Meanwhile, since NSGA-II is a multi-objective optimizer that focuses on solving the Pareto set of the optimal solution [27], it cannot guarantee a stable decrease of the fitness function during the iteration, which is not conducive to searching for the gluing region with an optimal fitness value. It indicates that IMWOA-EWM performs better in processing signals with strong background noise.

**Figure 6:**
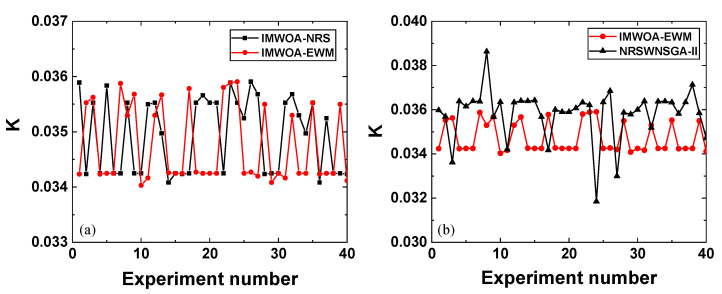
Stability of the fitting slope *K* for midday sample gluing. The red line plot with solid circles indicates the fitting slope obtained by the IMWOA-EWM method proposed in this study. The black lines represent the fitting slopes obtained by comparative methods, where the black solid squares corresponding to the IMWOA-NRS method and black solid triangles corresponding to the NRSWNSGA-II method. (**a**) Comparison between IMWOA-EWM and IMWOA-NRS. (**b**) Comparison between IMWOA-EWM and NRSWNSGA-II.

To verify the gluing stability of the IMWOA-EWM method, the fitting slopes gained by IMWOA-EWM in 40 replicates of the gluing experiment were counted and compared to those obtained by IMWOA-NRS and NRSWNSGA-II, as shown in Figure 6.

The variance of the fitting slope gained by the NRSWNSGA-II method is 1.31 × 10^−6^. The fitting slopes derived from the IMWOA-EWM and IMWOA-NRS methods both have a variance of 4.9 × 10^−7^, indicating better stability compared to NRSWNSGA-II. This improved stability is attributed to the stronger search capability of IMWOA and the excellent performance of the objective function *D*. The result also demonstrates that the EWM weighting method can be effectively applied to finding the optimal region without the need for massive amounts of training samples.

### 3.4. Full-Day Gluing Experiment

**Figure 7:**
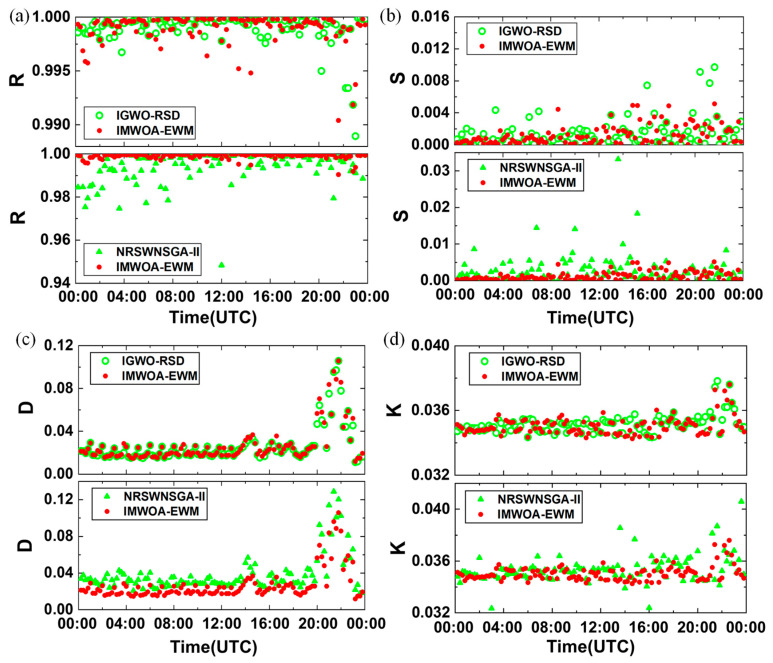
The full-day signal gluing experiments in Korea. Red solid dots indicate the results of the IMWOA-EWM method proposed in this study. Green symbols represent the results of comparative methods, where green hollow circles corresponding to the IGWO-RSD method and green solid triangles corresponding to the NRSWNSGA-II method. (**a**) Correlation coefficient *R*. (**b**) Regression stability coefficient *S*. (**c**) Mean fit deviation *D*. (**d**) Fitting slope *K*.

To comprehensively evaluate the performance of IMWOA-EWM under different signal quality conditions, this paper selected samples from the AHSRL system (University of Wisconsin, Madison, WI, USA) in Korea at different times throughout the day on May 8, 2017, for gluing experiments and conducted comparative analyses with IGWO-RSD and NRSWNSGA-II. The four indicators, including *R*, *S*, *D*, and the fitting slope *K*, were analyzed to compare the fitting results of the three algorithms. Figure 7 presents the results of the three algorithms. From the figure, it is evident that the gluing results from the NRSWNSGA-II method exhibit the most significant fluctuation, producing a minimum value of *R* = 0.948 with a variance of 5.476 × 10^−5^. At a 95% confidence level, the confidence interval for *R* is 0.9931–0.9958. In comparison, the IGWO-RSD method yields more stable results, with an average value of *R* = 0.999 and a confidence interval ranging from 0.9987 to 0.9993. The performance of the IMWOA-EWM method is even better, sharing the same confidence interval as the IGWO-RSD method, while achieving a minimum value of *R* = 0.9904. Regarding regression stability, smaller values of *S* denote more stable regression results. For IMWOA-EWM, all values of *S* are within 0.0051, with a confidence interval of 0.001–0.0015. For IGWO-RSD, the maximum value of *S* is 0.0097, with a confidence interval of 0.001–0.0017. While for NRSWNSGA-II, the maximum value of *S* is 0.0332, with a confidence interval of 0.002–0.0035. In terms of the dual-channel mean deviation *D*, the confidence interval of *D* by NRSWNSGA-II is 0.0344–0.0418, which is larger than the confidence intervals for IGWO-RSD and IMWOA-EWM (both 0.023–0.029). This indicates that NRSWNSGA-II has a greater deviation in the gluing results. As to the stability of the fitting slope *K*, its variance for the NRSWNSGA-II method is 7.29 × 10^−6^, while for IGWO-RSD and IMWOA-EWM, its variance is 3.6 × 10^−7^.

Considering the distributions and confidence intervals of the *R*, *S*, *D*, and *K* for the three algorithms, both IGWO-RSD and IMWOA-EWM demonstrate superior performance in accuracy and stability compared to the NRSWNSGA-II algorithm. Notably, IMWOA-EWM exhibits significantly higher computational efficiency than IGWO-RSD. Its average runtime for gluing full-day samples from Korea is 33.47 s, approximately half of IGWO-RSD’s runtime (62.35 s). Additionally, IMWOA-EWM shows a significant advantage over IGWO-RSD in the regression stability coefficient, owing to the increased weights of *S* obtained by the EWM method.

## 4. Discussion

**Figure 8:**
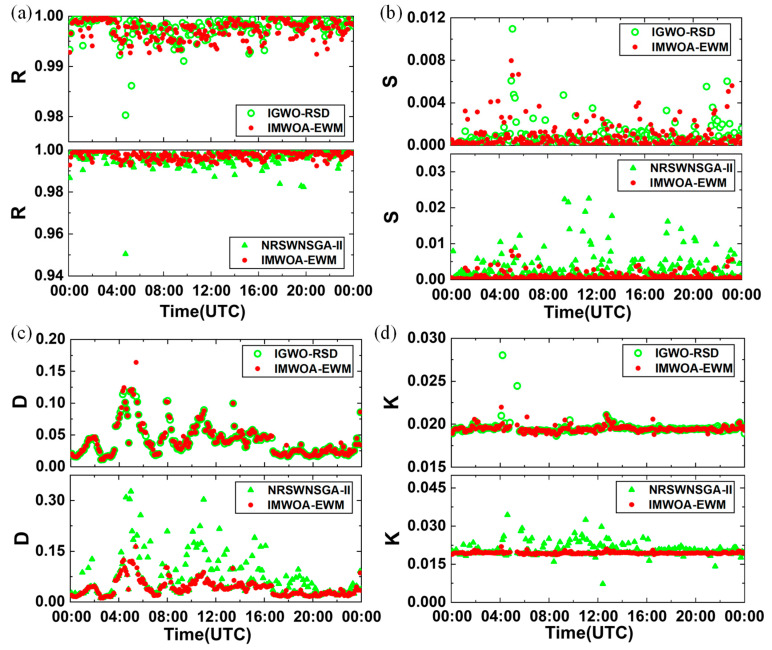
The full-day signal gluing experiments in Singapore. Red solid dots indicate the results of the IMWOA-EWM method proposed in this study. Green symbols represent the results of comparative methods, where green hollow circles corresponding to the IGWO-RSD method and green solid triangles corresponding to the NRSWNSGA-II method. (**a**) Correlation coefficient *R*. (**b**) Regression stability coefficient *S*. (**c**) Mean fit deviation *D*. (**d**) Fitting slope *K*.

Another full-day gluing experiment was conducted based on the data of July 5, 2013, in Singapore, as referenced in [18]. The comparison of the gluing results of the three algorithms is shown in Figure 8. It can be seen that the IMWOA-EWM method significantly outperforms NRSWNSGA-II and IGWO-RSD concerning the distribution of *R*, *S*, and *K*. Unlike the gluing results in Korea, although the IMWOA-EWM method is significantly better than NRSWNSGA-II in terms of mean deviation *D*, it is slightly inferior to IGWO-RSD. The maximum value of *D* for IGWO-RSD is 0.1198, while it is 0.164 for IMWOA-EWM. One possible explanation is that IGWO-RSD places greater emphasis on *D* in its weight allocation, thus achieving better performance regarding mean fit deviation. Nevertheless, despite the weights of the IMWOA-EWM method being trained based on the Korean sample data, it still demonstrated a good gluing effect in the Singaporean samples. Table 2 presents the statistics of *R*, *S*, and *D* derived from the samples used for weight allocation. Since the median absolute deviation method was utilized before executing the EWM method to remove outliers, the median values of *R*, *S*, and *D* for the Singaporean samples were calculated to be 0.9983, 1.2326, and 0.0155, respectively. These values were found to fall within the range defined by the median confidence interval of the Korean samples. Therefore, the weights obtained by EWM can be directly applied to other datasets without reallocation, when the data to be glued meet the median of *R*, *S*, and *D,* which is approximately within the ranges of 0.998–1, 1.228–3.746, and 0.011–0.02, respectively.

To further verify the effectiveness of IMWOA-EWM, this study employed measured signals obtained from airborne Rayleigh lidar detection experiments to conduct dual-channel gluing tests. The detection experiment was carried out over Huainan City, Anhui Province (116.8° E, 32.5° N) from 19:00 local time (LT) on March 31, 2024, to 05:00 LT on April 1, 2024. In the lidar system, the single-pulse energy of the laser was 26 mJ, the repetition frequency was 50 Hz, the laser wavelength was 532 nm, the diameter of the receiving telescope was 350 mm, and the working height of the lidar was 2.7 km. All measured profiles underwent preprocessing, including dead-time correction and trigger delay correction. Subsequently, 20 groups of measured signals from different time periods were randomly selected to statistically analyze the *R*, *S*, and *D* of the dual-channel signals of the Rayleigh lidar. The results showed that the median values of *R*, *S*, and *D* were 0.998, 2.04, and 0.012, respectively, all falling within the preset applicable ranges (*R*: 0.998–1, *S*: 1.228–3.746, *D*: 0.011–0.02). Therefore, the weights obtained by EWM could be directly used.

**Figure 9:**
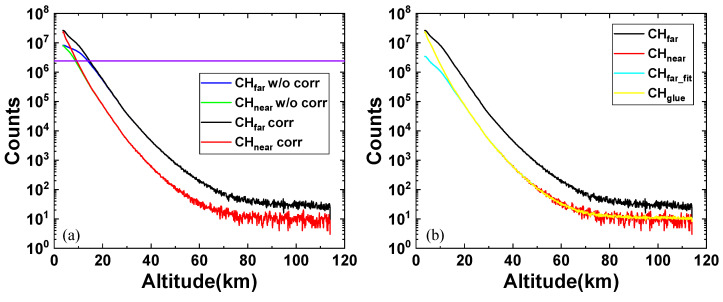
Dual-channel signal gluing for airborne Rayleigh lidar. Here, the blue and green curves represent the far-field and near-field channel signals before dead-time correction, respectively. The black and red curves show the far-field and near-field channel signals after dead-time correction, respectively. The purple horizontal line represents the maximum correctable value of the non-paralyzable model. The cyan curve is fitted from the far-field channel signal by IMWOA-EWM, and the yellow curve represents the glued signal. (**a**) Dual-channel data before and after dead-time correction. (**b**) Dual-channel gluing results.

Figure 9a presents the dual-channel data before and after dead-time correction with a 1 h integration time. The purple horizontal line represents the maximum correctable value of the non-paralyzable model. By combining the maximum correctable value with the SNR, the effective detection range of the far-field channel was determined to be 14.6 km–61.5 km, while the effective detection range of the near-field channel covered 9.3 km–48.3 km. After intelligently searching for the optimal gluing region by the IMWOA-EWM method, the resulting dual-channel gluing outcome (the yellow profile in Figure 9b) demonstrated significant advantages. It not only corrected the saturated nonlinearity of the far-field channel (black profile) below 14.6 km but also resolved the issue of excessively low SNR in the near-field channel (red profile) above 48 km. Table 3 lists the values of the four evaluation indicators (*R*, *S*, *D*, and *K*) calculated based on the aforementioned gluing results. These values reached relatively ideal levels, proving that IMWOA-EWM can be effectively applied to the gluing task of the Rayleigh lidar system. Meanwhile, the applicable conditions proposed in this study were also verified.

## 5. Conclusions

An efficient signal gluing method for dual-channel atmospheric lidar is developed, named IMWOA-EWM, which integrates the improved whale optimization algorithm (IMWOA) and the entropy weight method (EWM). Here, IMWOA was employed to optimize the fitness function, which can significantly improve computational efficiency compared with IGWO. The fitness function of IMWOA was composed of *R*, *S*, and *D*, with the corresponding weights allocated by EWM being 0.3952, 0.2984, and 0.3064, respectively. EWM does not demand excessive training samples and considerably reduces computational load. Based on ground-based aerosol lidar data, the runtime, solving capability, gluing accuracy, and stability of IMWOA-EWM were verified. Moreover, through full-day signal gluing experiments, the transferability and applicable conditions of the weights in IMWOA-EWM were discussed. The results demonstrate that IMWOA-EWM can accurately identify the optimal signal gluing region, thanks to the IMWOA algorithm’s excellent global search capability and the higher weight assigned to the objective function *S* by the EWM method. It can increase the correlation coefficient *R* of the dual-channel gluing signal to above 0.99, with a confidence interval of 0.9987–0.9993 at a 95% confidence level, outperforming IGWO-RSD and NRSWNSGA-II. In terms of gluing quality, IMWOA-EWM yields more stable signals, especially for full-day signals from Korea. Its regression stability coefficient *S* is reduced by 47.4% and 84.6% compared to the IGWO-RSD and NRSWNSGA-II methods, respectively, with a confidence interval of 0.001–0.0015. Meanwhile, the IMWOA-EWM method has a good mean fit deviation *D*, with a confidence interval of 0.023–0.029. The IMWOA-EWM method also demonstrates good robustness and computational efficiency, with a runtime for a single gluing task being only half that of IGWO-RSD. Additionally, the weights obtained by EWM can be directly applied to other datasets without reallocation, provided that the median values of *R*, *S*, and *D* in the data to be glued fall approximately within the ranges of 0.998–1, 1.228–3.746, and 0.011–0.02, respectively. It is believed that the IMWOA-EWM method holds great value in large-scale lidar signal real-time processing, providing an effective and high-quality solution for the signal gluing of dual-channel atmospheric lidar.

## Figures and Tables

**Table 1:** Operating parameters of IMWOA-EWM.

Parameter	Value	Parameter	Value
Number of search agents	50	Number of iterations	50
Weight of *R*	0.3952	The SNR for the highest point	10
Weight of *S*	0.2984	Time resolution of samples	1 h
Weight of *D*	0.3064	Vertical resolution of samples	7.5 meter

**Table 2:** The statistics of *R*, *S*, and *D* derived from the samples used for allocating weights in EWM.

Objective Function	Minimum	Maximum	Median	Median Confidence Interval
*R*	0.726	1	0.9992	0.998–1
*S*	0.0001	28.47	2.55	1.228–3.746
*D*	0.0063	195.72	0.014	0.011–0.02

**Table 3:** Evaluation of dual-channel signal gluing results from airborne Rayleigh lidar.

Evaluation Indicators	Value
*R*	0.9986
*S*	0.0023
*D*	0.0304
*K*	0.1313

Note: The airborne Rayleigh lidar was developed by our research group, which is affiliated with Tianjin University in Tianjin, China.

## Data Availability

The datasets presented in this article are not readily available because the data are part of an on-going study. Requests to access the datasets should be directed to the corresponding author.
